# 809 An Allogenic Fat-First Approach to Burn Reconstruction Mitigates Adhesion and Soft Tissue Deficit

**DOI:** 10.1093/jbcr/irac012.357

**Published:** 2022-03-23

**Authors:** Shawn J Loder, Phoebe Lee, Matthew Cannon, Patricia Leftwich, Lauren Kokai, Kacey Marra, J Rubin

**Affiliations:** University of Pittsburgh, Pittsburgh, Pennsylvania; University of Pittsburgh, Pittsburgh, Pennsylvania; University of Pittsburgh, Pittsburgh, Pennsylvania; University of Pittsburgh, Pittsburgh, Pennsylvania; University of Pittsburgh, Pittsburgh, Pennsylvania; University of Pittsburgh, Pittsburgh, Pennsylvania; University of Pittsburgh, Pittsburgh, Pennsylvania

## Abstract

**Introduction:**

Adipose and adipose-derived stem cell therapies have met success as adjunctive treatment during burn reconstruction with well described benefit in the delayed-treatment of soft-tissue deficits. While the use of allogeneic skin is well-described, adipose tissues have typically remained autologous. Allogenic fat is not commonly used in burn care, however, in large, complex burns where autologous tissue is limited adipose may not be readily available for harvest or use. Understanding the efficacy of allogeneic tissues in this setting is critical to expand our reconstructive options. Here we describe a protocol utilizing allogeneic fat as well as examine the efficacy of this approach on burn-wound contractures, adhesions, and soft-tissue deficits.

**Methods:**

Female, Yorkshire swine received 16, 4x4 cm full-thickness burns. After 48 hours, eschar was removed to fascia. Wounds were stratified to receive either A) No Reconstruction, B) Skin-Only, C) Fat-Only, D) Immediate-Skin, Delayed-Fat, or E) Immediate-Fat, Delayed-Skin. All fat utilized was allogeneic sourced from vendor-matched swine. At 8-weeks post-engraftment animals were sacrificed and all wounds were collected for photography, ultrasound, histology and serum studies.

**Results:**

Use of allogeneic fat significantly improved terminal soft-tissue thickness under both immediate and delayed administration (p< 0.05). Immediate use of allogeneic fat significantly improved tissue mobility vs. untreated and skin graft controls (p< 0.05). Contracture was most significantly affected by timing of skin graft placement, however, could be further mitigated under standard delayed-fat approached with allogeneic tissue.

**Conclusions:**

Here we demonstrate use of allogeneic fat in both traditional-delayed and a fat-first approach with significant mitigation of adhesion when applied as an initial basal layer. Both immediate and delayed allogeneic fat were sufficient to improve on soft tissue deficits.

